# lncRNA MALAT1/miR-143 axis is a potential biomarker for in-stent restenosis and is involved in the multiplication of vascular smooth muscle cells 

**DOI:** 10.1515/biol-2021-0126

**Published:** 2021-12-20

**Authors:** Chen Cao, Wei Zhen, Haibin Yu, Li Zhang, Yiling Liu

**Affiliations:** Interventional Department, The Second Affiliated Hospital, Zhengzhou University, Zhengzhou 450014, China; President’s Office, The Second Affiliated Hospital, Zhengzhou University, Zhengzhou 450014, China; Nursing Department, The Second Affiliated Hospital, Zhengzhou University, Zhengzhou 450014, China; Department of Otorhinolaryngology, The Second Affiliated Hospital, Zhengzhou University, Zhengzhou 450014, China

**Keywords:** MALAT1, miR-14, in-stent restenosis, cell multiplication, cell invasiveness

## Abstract

The purpose of this study is to observe the potential value and underlying mechanism of the metastasis-associated lung adenocarcinoma transcript 1 (MALAT1)/miR-143 axis in ISR. A total of 150 participants were enrolled, including 100 patients (observation group) with coronary heart disease who underwent stent implantation in the Department of Cardiology of our hospital between January 2018 and January 2020, and 50 healthy people (control group) concurrently underwent a physical examination. Serum MALAT1 and miR-143 levels were detected by quantitative reverse transcription-polymerase chain reaction (qRT-PCR). Tumor necrosis factor-α (TNF-α; 10 ng/mL) induced human vascular smooth muscle cells (HVSMCs). MALAT1 increased while miR-143 decreased in the observation group versus the control group (*P* < 0.001). The non-restenosis group had significantly elevated MALAT1 expression while decreased miR-143 expression than the restenosis group (*P* < 0.001). The areas under the curves of the expression of MALAT1 and miR-143 in predicting restenosis were 0.917 and 0.881, respectively. Following si-MALAT1 transfection, HVSMC multiplication and invasiveness decreased significantly (*P* < 0.05). miR-143-inhibitor was observed to upregulate the luciferase activity of MALAT1-WT (*P* < 0.05). MALAT1 is highly expressed in patients with ISR while miR-143 is decreased, and the MALAT1/miR-143 axis is a potential pathway to modulate the multiplication and invasiveness of HVSMCs.

## 1 Introduction

Coronary heart disease (CHD), also known as coronary atherosclerotic heart disease, is a common cardiovascular disease and a global public health safety issue at present [1,2]. The latest statistics reveal a global incidence of CHD as high as 36.37% [3]. Coronary stent implantation, as an important intervention for heart rate rhythm, is mainly to improve the myocardial blood supply by reconstructing the narrow lumen to achieve the purpose of treatment [4]. However, in-stent restenosis (ISR) after stent implantation has always been one of the difficulties in coronary interventional therapy. Despite the significantly reduced incidence of ISR, thanks to the advent of drug-coated stents in recent years, ISR cannot be avoided entirely [5,6].

Long-chain noncoding RNAs (lncRNAs) are noncoding RNAs of over 200 nt in length, which cannot directly encode proteins [7,8]. Although the bulk of the human genome is translated into RNA molecules, only around 2% of these transcripts encode proteins. As a result, scientists have begun to investigate the vast universe of noncoding RNAs (ncRNAs) as critical physiological and pathological cell activity regulators. Numerous studies have shown the role of small noncoding RNAs (ncRNAs) such as microRNAs (miRNAs) in cardiovascular illness. Still, the role of long noncoding RNAs (lncRNAs) as important regulators in the genesis and progression of cardiac disorders remains unknown. Because all ncRNAs with a length of more than 200 nucleotides are arbitrarily classed as lncRNAs, this class of molecules is highly diverse, performing a range of biological activities and interacting with a variety of other RNAs and proteins [9]. Nonetheless, recent studies have found that lncRNAs are involved in many malignant tumors [10,11] and interfere with the occurrence and progression of a broad spectrum of cardiovascular diseases and nervous system diseases [12,13]. Importantly, lncRNAs have also been shown to play essential roles in embryonic development and cardiac lineage commitment (such as Fendrr and Braveheart [14]), and are important regulators of proper heart functionality. More details on lncRNAs in organogenesis and heart development have been extensively reviewed elsewhere [15]. Of these, metabolism associated lung adenocarcinoma transcript 1 (MALAT1), a highly conserved lncRNA located on chromosome 11q13.1 [14], is of predictive value in vascular stenosis of CHD patients [15], despite the fact that it cannot directly encode proteins as reported by the previous literature [16]. In addition, in recent years, lncRNAs have been revealed to be able to regulate microRNAs (miRs) by acting as competitive endogenous RNAs (ceRNAs) to participate in a variety of diseases [17]. For example, the *MALAT1* has vital functions in nuclear speckles and the regulation of genes expressions. Recent research studies have proved that *MALAT1* was overexpressed and oncogenic in some tumors, including lung, colorectal, bladder, and laryngeal cancers [18]. MALAT1/miR-15b-5p/MAPK1 axis mediates endothelial progenitor cell autophagy via the mTOR pathway and affects coronary atherosclerotic heart disease [19]. miR-31 promotes Th22 differentiation in patients with CHR by targeting Bach2 [20], and lncRNA ANRIL and miR-181b mediate the interaction of the NF-kappaB axis in inflammation-related coronary artery disease [21]. miR is a kind of 22 nt-length long noncoding short-chain RNAs in eukaryotes, which can bind to 3′UTRs and 5′UTRs of downstream target genes and coding area to regulate the expression of target genes and participate in many intercellular signal regulation [22]. miR-143, a classic miR, has been revealed to be pivotal in the occurrence and development of CHR via modulating KLLN and its mechanism [23]. Another literature study has pointed out that miR-143 is an independent, prognostic, and diagnostic marker of CHR [24]. In this study, we found that there were potential targeting sites between lncRNA MALAT1 and miR-143 through online prediction.

Hence, we speculate that the two may be potential predictors of ISR, with certain regulatory mechanisms between them.

## 2 Methods and materials

### 2.1 Clinical data

One hundred CHR patients (observation group, hereinafter referred to as OG) who received stent implantation in the Department of Cardiology of our hospital from January 2018 to January 2020 and 50 healthy controls (control group, hereinafter referred to as CG) without CHR who concurrently underwent physical examination were collected as research participants. Gender and age were similar in the two cohorts (*P* > 0.05).

#### 2.1.1 Inclusion criteria for patients

Patients between 55 and 75 years of age who were tolerant to stent implantation were included. Patients in OG were all diagnosed with CHR by coronary angiography.

#### 2.1.2 Exclusion criteria for patients

Exclusion criteria included severe liver and kidney dysfunction, other infectious or immune diseases, communication disorder, cognitive dysfunction, and malignant tumor(s).


**Informed consent:** Informed consent has been obtained from all individuals included in this study.
**Ethical approval:** The research related to human use has been complied with all the relevant national regulations, institutional policies and in accordance with the tenets of the Helsinki declaration, and has been approved by the author's institutional review board or equivalent committee.

### 2.2 Cell source

Human vascular smooth muscle cells (HVSMCs) were purchased from Beijing Bena Culture Collection (BNCC340087). Cells were cultured in Dulbecco’s modified Eagle’s medium (DMEM) with 10% fetal bovine serum (FBS), in an incubator supplemented with 5% CO_2_ at 37°C and were passaged when the confluence reached 80%.

### 2.3 Cell transfection

HVSMCs were transfected with si-MALAT1 (inhibitory sequence), si-NC (independent sequence; control), miR-143-inhibitor (inhibitory sequence), and miR-NC (control) with the Lipofectamine 2000 transfection kit according to the kit’s instructions. All the sequences were designed and synthesized by Shanghai Sangon Biological Co., Ltd. Then, the cells were transfected by tumor necrosis factor-α (TNF-α; 10 ng/mL) for 24 h. The transfection effect was verified by quantitative reverse transcription-polymerase chain reaction (qRT-PCR).

### 2.4 qRT-PCR detection

The total RNA was separated from collected serum and cells with TRIzol reagent and subjected to purity, concentration, and integrity determination using an ultraviolet spectrophotometer and gel electrophoresis. cDNA was then obtained using the reverse transcription kit. Subsequently, PCR amplification of miRs was carried out with an all-in-one miRNA first-chain cDNA synthesis kit and all-in-one miR qPCR kit, while that of lncRNA and mRNA was done by SYBR Premix Ex Taq kits. RT-qPCR (ABI 7500, ABI) instrument was used for detection. Amplification conditions were as follows: predenaturation at 95°C for 1 min, followed by 40 cycles of 95°C for 15 s and 60°C for 1 min; 95°C for 15 s, 60°C 1 min, and finally, 95°C for 15 s for melting curve analysis. The amplification system was configured according to the kit instructions. U6 and GAPDH were used as an internal reference for miR and lncRNA, respectively, and the target genes’ expression profiles were calculated using the 2^−ΔΔCT^ method [25]. The amplified primer sequences are detailed in Table 1.

**Table 1:** Primer sequences

Gene name	Upstream sequence 5-3	Downstream sequence 5-3
MALAT1	GCCTGGAAGCTGAAAAACGG	TGGAAAACGCCTCAATCCCA
miR-143	AGTGCGTGTCGTGGTGT	GCCTGAGATGAAGCACGTG
GAPDH	CTGACTTCAACAGCGACACC	GTGGTCCAGGGGTCTTACTC
U6	TGCGGGTGCTCGCTTCGGCAGC	CCAGTGCAGGGTCCGAGGT

### 2.5 Determination of cell multiplication

This study analyzed cell viability using the cell counting kit 8 (CCK-8) (Dojindo Molecular Technologies, Inc., Kumamoto, Japan). Cells (4 × 10^3^ cells per well) seeded into the wells of a 96-well plate were cultivated at 37°C with 5% CO_2_ in the air and then immersed in a CCK-8 reagent (10 µL) at 0, 24, 48, and 72 h after cultivation, respectively. After another 2 h of incubation at 37°C, the cells were processed with a microplate reader for optical density (450 nm) determination.

### 2.6 Detection of cell invasiveness

Transwell test was used to detect the changes in cell invasiveness capacity. After 48 h of transfection, 200 μL of cell suspension (1 × 10^8^/L) was put into the upper chamber containing Matrigel (Corning, USA), and 500 μL of DMEM containing 10% FBS was added into the lower chamber for 24 h of culture in the cell incubator. Then, the chamber was subjected to 3 PBS rinses, 10 min of fixing with formaldehyde, and 15 min of dyeing with 0.1% crystal violet solution. After washing 3 times with PSB, the cells inside the ventricular membrane were gently wiped off with cotton swabs, and the chamber was taken off and laid on the slide to count the invasive cells in three visual fields under a microscope (200×).

### 2.7 Double luciferase reporter assay (DLRA)

miRs that could bound to MALAT1 were predicted using online prediction websites StarBase v3.0 and LncBase Predicted v.2, and it was found that there were targeted binding sites between MALAT1 and miR-143. Then, the wild-type (WT) and mutant (MUT) MALAT1 luciferase reporter plasmid vectors were constructed. HVSMCs were inoculated into a 24-well plate with 1 × 10^5^ cells per well and co-transfected with either miR-NC or miR-143-inhibitor after 24 h. DLRA was conducted 24 h later to detect the activity of Renilla and firefly luciferases, with Renilla luciferase as an internal reference. The experiment was repeated 3 times.

### 2.8 Statistical analysis

In this study, GraphPad 8 software package was adopted to draw the required pictures, and the SPSS20.0 software package was used to conduct statistical analysis on the collected data. Inter-group comparisons employed independent sample t-test, multigroup comparisons adopted one-way ANOVA (expressed as F) and LSD/t posthoc test, and multitime point expression comparisons used repeated measures ANOVA (expressed as F) and Bonferroni posthoc test. The correlation analysis was performed by Pearson correlation coefficient, and the diagnostic value of lncRNA and miR in restenosis patients was visualized using receiver operating characteristic (ROC) curves. A *P* value less than 0.05 was considered statistically significant.

## 3 Results

### 3.1 Expression and predictive values of MALAT1 and miR-143 in patients with restenosis

**Figure 1:**
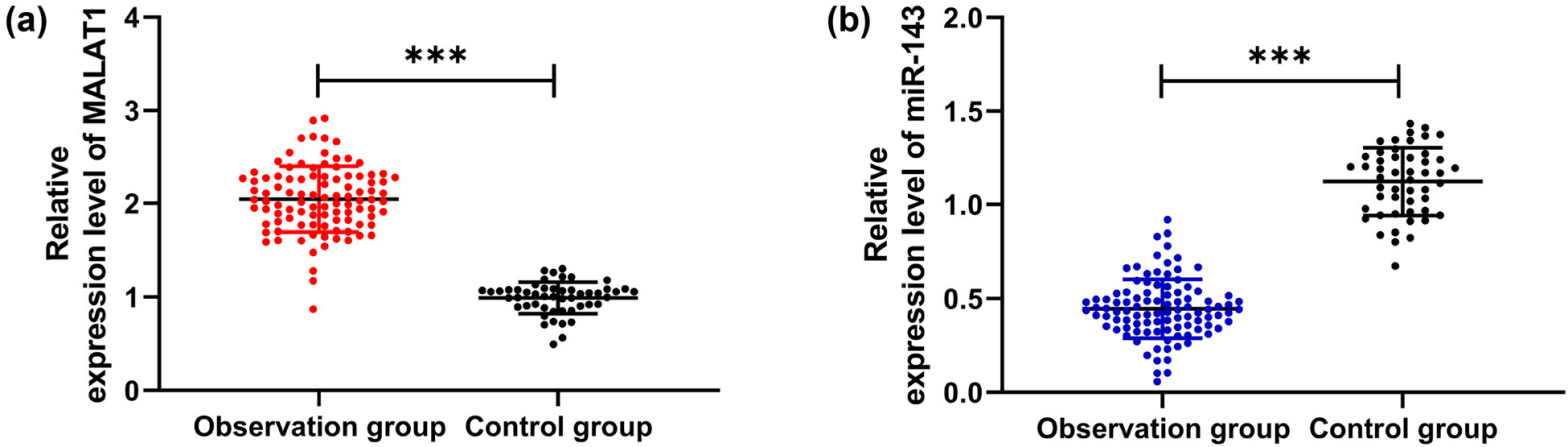
Expression of MALAT1 and miR-143 in CHD patients. (a) QRT-PCR was used to detect the expression level of MALAT1 in the serum of healthy people and CHD patients (*n* = 50). (b) QRT-PCR was used to detect the expression level of miR-143 in the serum of healthy people and CHD patients (*n* = 50). ***P < 0.001.

**Figure 2:**
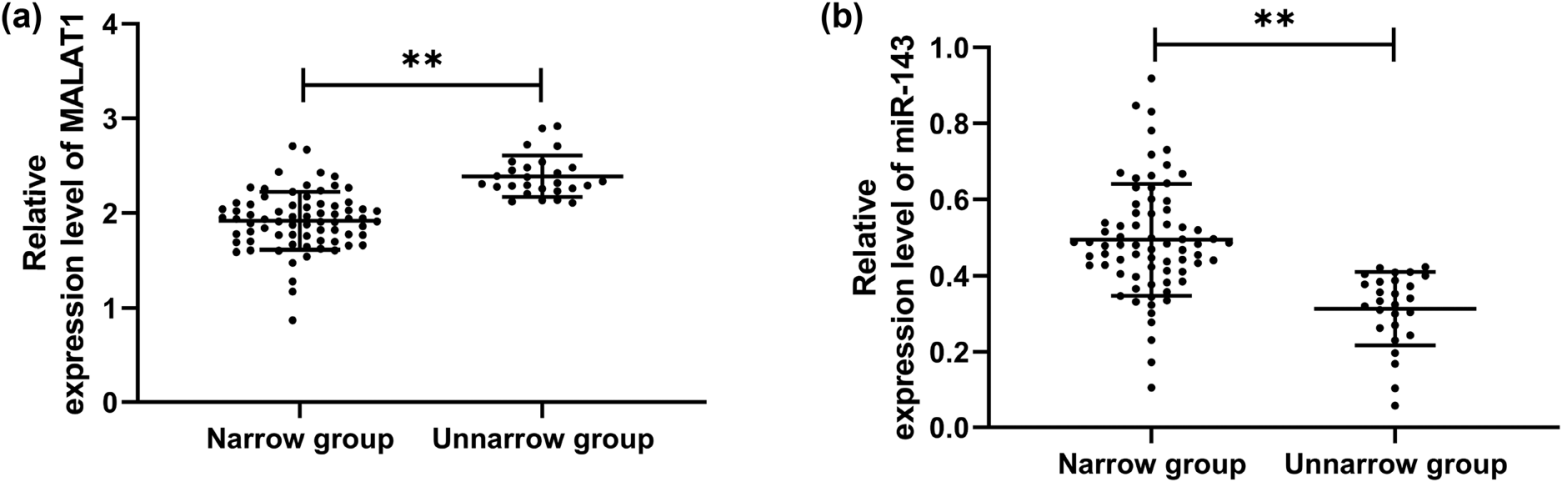
Expression of MALAT1 and miR-143 in CHD patients with restenosis. (a) Expression of MALAT1 in CHD patients with restenosis by qRT-PCR (restenosis group = 27, nonrestenosis group = 73). (b) Expression of miR-143 in CHD patients with restenosis by qRT-PCR (restenosis group = 27, nonrestenosis group = 73). **P < 0.01.

**Figure 3:**
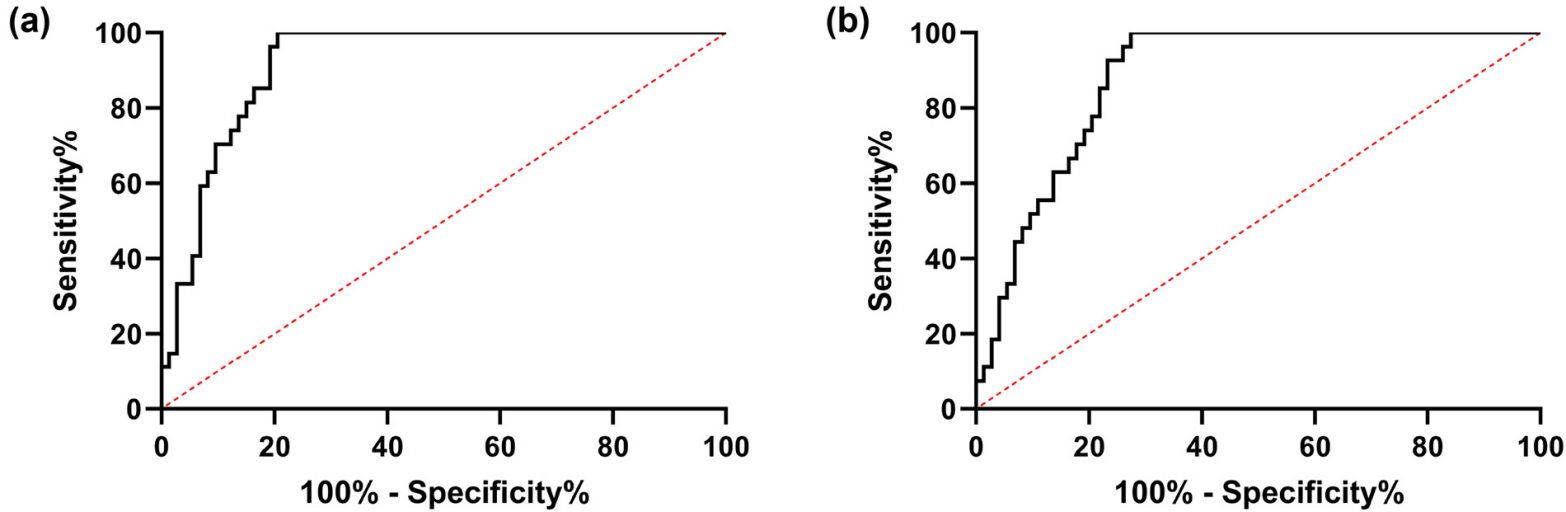
Predictive value of MALAT1 and miR-143 in CHD patients with restenosis. (a) ROC curve analysis of the AUC of MALAT1 in predicting restenosis in patients with CHD. (b) ROC curve analysis of the AUC of miR-143 in predicting restenosis in patients with CHD.

To explore the expression of MALAT1 and miR-143 in patients with stenosis, we first analyzed their levels in CHD patients. It was found that MALAT1 increased while miR-143 decreased in OG compared with CG (Figure 1a and b, *P* < 0.001), indicating that the two interfered with the development of CHD. To further study the correlation of MALAT1 and miR-143 with restenosis in CHD patients, we divided 100 patients into the restenosis group (*n* = 27) and nonrestenosis group (*n* = 73) according to the stenosis status after treatment to further detect the expression of MALAT1 and miR-143 in patients. By testing, we found that MALAT1 was significantly increased, and miR-143 was decreased in the restenosis group compared with the nonrestenosis group (Figure 2a and b, *P* < 0.001). Then, we analyzed the predictive value of the two in patients with restenosis using the ROC curve. It showed that the areas under the curves (AUCs) of MALAT1 and miR-143 were 0.917 and 0.881, respectively (Figure 3 and Table 2), suggesting that the two were of high predictive value in patients with restenosis.

**Table 2:** ROC parameters

Variables	AUC	95 CI%	Specificity (%)	Sensitivity	Youden index (%)	Cutoff value
MALAT1	0.917	0.864–0.989	79.45	100.00%	79.45	>2.108
miR-143	0.881	0.818–0.945	72.60	100.00	72.60	<0.426

Note: AUC: area under the curve, 95 CI%: 95% confidence interval.

### 3.2 Downregulation of MALAT1 suppresses the multiplication and invasiveness of HVSMCs

**Figure 4:**
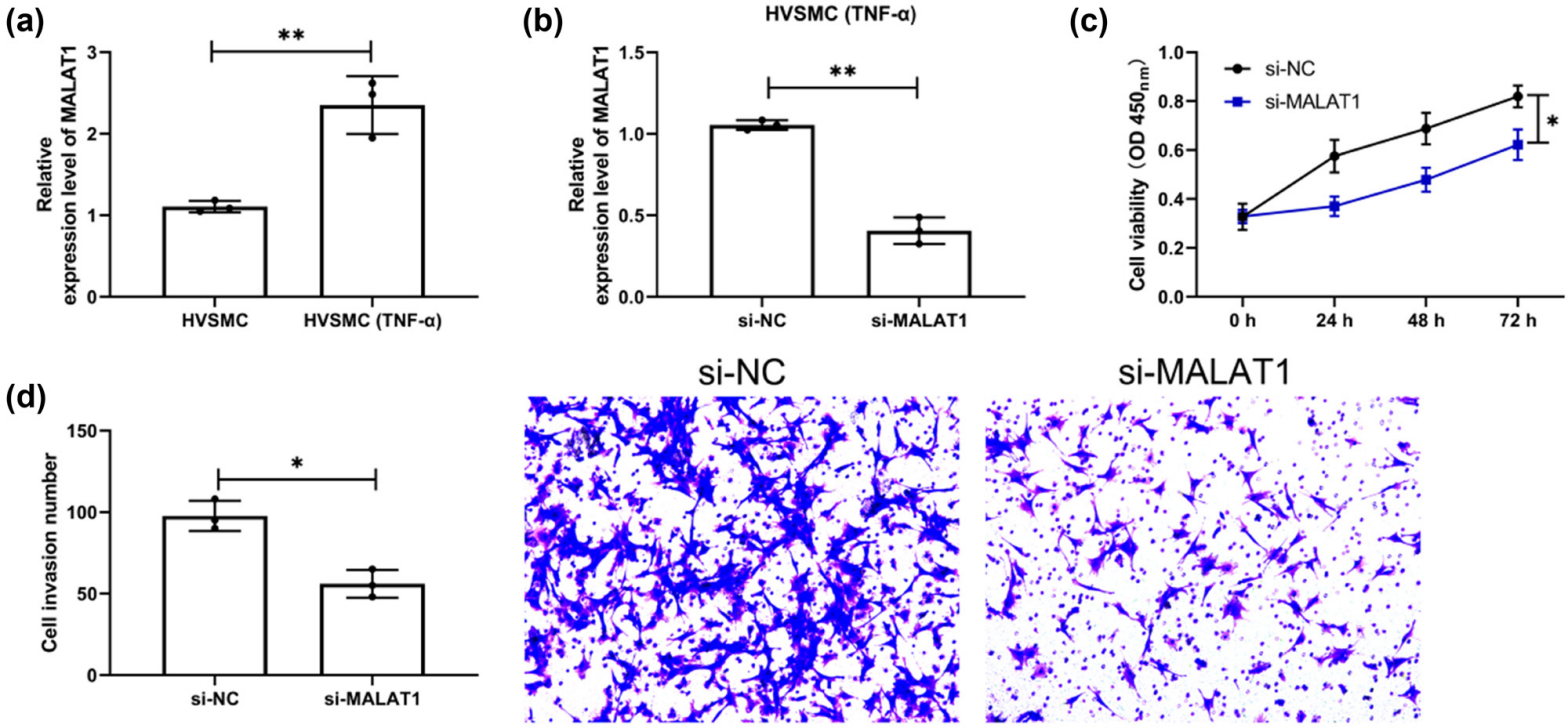
Downregulation of MALAT1 inhibits the multiplication and invasiveness of HVSMCs. (a) QRT-PCR was used to detect the expression level of MALAT1 in the cells after TNF-α-induced HVSMCs. (*n* = 3). (b) QRT-PCR was used to detect the expression level of MALAT1 in HVSMCs cells induced by TNF-α after si-MALAT1 transfection (*n* = 3). (c) Changes in multiplication ability of si-MALAT1-transfected HVSMCs after TNF-α induction by CCK-8 assay (*n* = 3). (d) Changes in invasiveness ability of si-MALAT1-transfected HVSMCs after TNF-α induction by Transwell assay (*n* = 3). *P < 0.05 and **P < 0.01.

Through the above, we confirmed the clinical value of MALAT1 and miR-143 in patients with restenosis. However, we are not clear about the related mechanism, so we treated HVSMCs with TNF-α for exploration. Through qRT-PCR detection, we found that MALAT1 elevated significantly in HVSMCs after intervention (Figure 4a, *P* < 0.01). Subsequently, si-MALAT1 was transfected into the intervened HVSMCs and remarkably reduced MALAT1 was determined (Figure 4b, *P* < 0.01). In addition, compared with HVSMCs treated with TNF-α after transfection of si-NC, the multiplication and invasiveness of HVSMCs treated with TNF-α after transfection of si-MALAT1 reduced evidently, as demonstrated by CCK-8 and Transwell assays (Figures 4c and d, *P* < 0.05).

### 3.3 MALAT1 has a targeted binding relationship with miR-143

**Figure 5:**
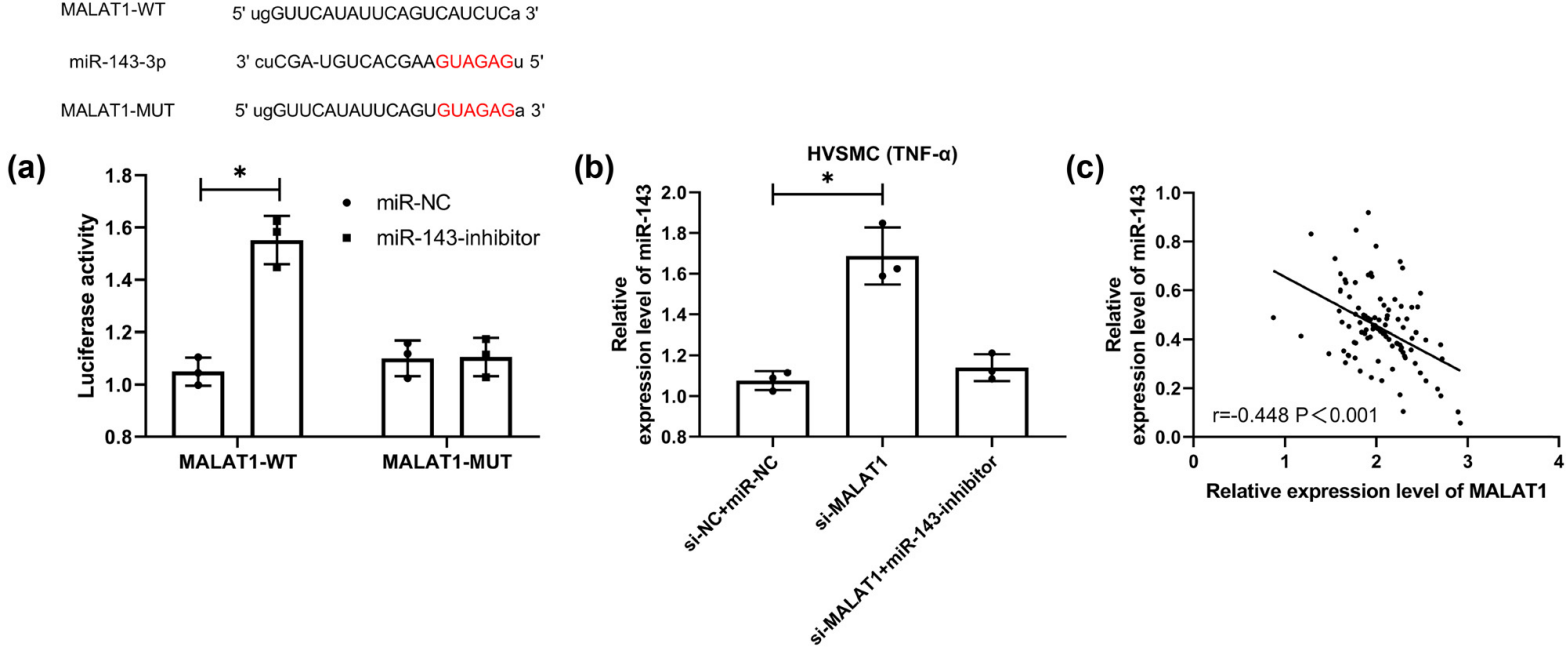
Targeted binding relationship between MALAT1 and miR-143. (a) The online prediction website verifies the target prediction of MALAT1 and miR-143 and the dual-luciferase report (*n* = 3). (b) qRT-PCR was used to detect the expression level of miR-143 in co-transfected si-MALAT1 cells (*n* = 3). (c) Correlation analysis of MALAT1 and miR-143 in CHD patients by Pearson correlation coefficient (*n* = 50). *P < 0.05.

CeRNA is an important way for lncRNA to participate in various functions of the body. To further determine the correlation between MALAT1 and miR-143, we conducted the DLRA. The experimental results showed that the miR-143-inhibitor could upregulate the luciferase activity of MALAT1-WT (Figure 5a, *P* < 0.05). Furthermore, qRT-PCR showed that miR-143 in cells transfected with si-MALAT1 was significantly increased but that in cells co-transfected with miR-143-inhibitor and si-MALAT1 was reversed (Figure 5b, *P* < 0.05). Moreover, an inverse association was determined between MALAT1 and miR-143 in CHD patients (Figure 5c). These experimental results indicate that MALAT1 has a targeted binding relationship with miR-143.

### 3.4 Downregulation of miR-143 reverses the inhibitory action of si-MALAT1 on multiplication and invasiveness of HVSMCs

**Figure 6:**
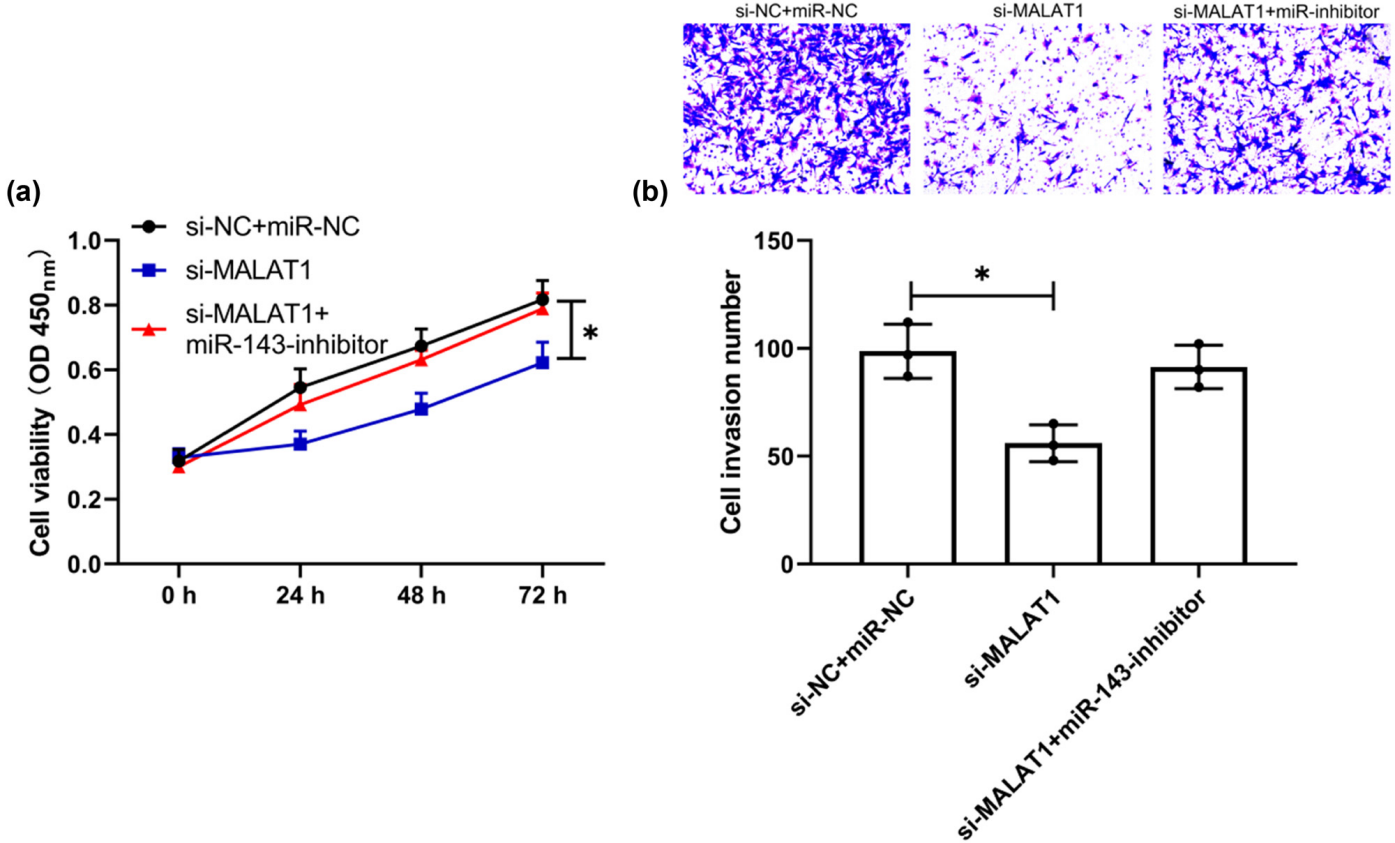
Downregulation of miR-143 reverses the inhibition of si-MALAT1 on the multiplication and invasiveness of HVSMCs. (a) Changes of cell multiplication ability after co-transfection by CCK-8 assay (*n* = 3). (b) Changes in cell invasiveness ability after co-transfection by Transwell assay (*n* = 3). *P < 0.05.

At the end of the study, the relationship between MALAT1 and miR-143 was further verified. The changes of multiplication and invasiveness of TNF-α-treated HVSMCs were analyzed by the co-transfection experiment. CCK-8 and Transwell experiments demonstrated that, after co-transfection of miR-143-inhibitor and si-MALAT1, the inhibitory action of si-MALAT1 on HVSMC multiplication and invasiveness was reversed (Figure 6a and b, *P* < 0.05). Therefore, the MALAT1/miR-143 axis can regulate the multiplication and invasiveness of HVSMCs and may be a potential therapeutic target.

## 4 Discussion

Recent years have witnessed the great progress made in the treatment of CHD, a commonly seen cardiovascular disease in clinical practice [26]. Currently, coronary stent implantation is the mainstay of treatment for CHR [27]. However, some patients still suffer from vascular restenosis after treatment, which is one of the causes of death in patients [28]. In this study, we found that MALAT1 and miR-143 were differentially expressed in CHD patients with restenosis, and MALAT1 can participate in the multiplication and invasiveness of HVSMCs via modulating miR-143.

lncRNAs have been reported to play an important regulatory role in many diseases, especially in metabolic diseases and cardiovascular diseases. For example, the research by Liu et al. proposed that insulin resistance of type 2 diabetes can be effectively reduced by the lncRNA MALAT1/miRNA-382-3p/resistin axis [29]. In addition, lncRNA CAIF is shown to suppress autophagy and alleviate myocardial infarction by blocking p53-mediated cardiac troponin transcription [30], and lncRNA H19 can reduce myocardial injury and maladaptive cardiac remodeling caused by myocardial infarction by regulating KDM3A [31]. MALAT1, an early discovered lncRNA, is found to be highly expressed in many cancers such as lung cancer [32], gastric carcinoma [33], and breast cancer [34]. CeRNA is currently one of the important ways for lncRNA to participate in the life process [35]. lncRNAs can also participate in the occurrence of acute myocardial infarction by regulating the downstream binding of miRs. For example, MALAT1 knockout can alleviate acute myocardial infarction through the miR-320/Pten axis [36], and lncRNA Gas5 targets the miR-525-5p/CALM2 axis to regulate myocardial infarction [37]. Earlier, a literature study has reported that miR-143 is involved in the development of CHD [38]. In this paper, through the database prediction analysis, we found the binding target sites between miR-143 and MALAT1, indicating a regulatory relationship between the two.

We first analyzed the expression of miR-143 and MALAT1 in CHD patients and found decreased expression of miR-143 and increased expression of MALAT1. Further, we detected miR-143 and MALAT1 in patients with restenosis and observed that MALAT1 was increased while miR-143 was decreased. Similarly, early studies found that MALAT1 was increased in patients with ISR, which verified the consistency of MALAT1 expression [14]. Furthermore, we detected the expression of miR-143. It was found for the first time that miR-143 was decreased in patients with ISR; moreover, ROC curve analysis showed that miR-143 had clinical value in predicting ISR. The above results suggest that miR-143 has a high clinical value for ISR in CHD patients but the underlying mechanism needs further verification.

The primary mechanism of ISR is an aggregation of platelets, leukocytes, and macrophages, which results in the migration and proliferation of medial smooth muscle cells [39,40]. Recently, researchers discovered that around one-third of ISR events were caused by in-stent neoatherosclerosis (ISNA), a mechanism that is clearly distinct from the traditional process [41]. However, there is a common phenomenon that occurs in each of these mechanisms: ISR and ISNA inflammation. Mir-143/145 has been demonstrated to be abundantly and selectively expressed in vascular smooth muscle cells (VSMCs) and to have a critical role in the proliferation and migration of VSMCs and the function of arteries, in earlier research [42,43]. Additional research established a direct link between miR-143/145 and cardiovascular illness in humans and experimental animal models [44,45]. miR-143/145 was demonstrated to be downregulated during carotid artery neointimal development in rats. Researchers discovered a substantial reduction in miR-143/145 expression in animal models of damaged or atherosclerotic arteries, including ApoE mutant mice’s atherosclerotic aortas, mouse ligation-induced carotid artery, and rat carotid balloon-injured carotid artery [44]. These data imply that miR-143/145 plays a role in developing atherosclerosis and ISR induced by VSMC migration and proliferation. Additionally, a few recent studies discovered that miR-143/145 might play some inflammatory disorders [46]. From the above experiments, we can see that the relative expression of MALAT1 and miR-143 was opposite in CHD patients with ISR, so we speculated that there might be a targeted regulation relationship between the two. It was identified by DLRA that MALAT1-WT fluorescence activity could be upregulated by miR-143-inhibit, and correlation analysis revealed an inverse connection between MALAT1 and miR-143 in CHD patients, which suggested a targeted regulatory relationship between them. Then, we found through experiments that the multiplication and invasiveness of TNF-α interfered HVSMCs was significantly inhibited after MALAT1 knockdown. However, after the co-transfection of the miR-143-inhibitor and si-MALAT1 into HVSMCs, the multiplication and invasiveness of cells were not significantly different from those transfected with miR-NC + si-NC. This suggests that the MALAT1/miR-143 axis may be a potential pathway for restenosis.

Experiments have determined the clinical value and relevant mechanism of the MALAT1/miR-143 axis in CHD patients with ISR. However, this study still shows margins of improvement. First, an in-depth investigation of the MALAT1/miR-143 axis mechanism in CHD patients with ISR is warranted. Second, more experiments are needed to verify the results since we only tested the impacts of the MALAT1/miR-143 axis on HVSMC multiplication and invasiveness. Therefore, we hope that we will refine our experimental design to improve our research conclusions in future research.

To sum up, MALAT1 is highly expressed in CHD patients with ISR while miR-143 expression is decreased. Besides, the MALAT1/miR-143 axis can be used as a potential pathway to modulate the multiplication and invasiveness of HVSMCs.

There are several limitations to this study. We used a limited number of cell lines as well as we did not evaluate the effect on the primary cells. However, some restrictions should garner more attention. For instance, the mTOR signaling pathway was not the only one active in CAD blood samples, as seen by the dot plot of the KEGG pathway. Although most of the genes involved in the mTOR signaling pathway were discovered in CAD, the other two pathways require more investigation. Additional *in vivo* investigations are required.

The more vigorous study needs to include numerous cells lines and human primary cell lines. The area of lncRNAs as possible therapeutic targets is still in its infancy. It is not implausible that lncRNAs may emerge as useful new tools for the treatment of a variety of illnesses, including CVDs, in the near future, which is worthy of future investigation. The discovery of lncRNAs revolutionized our knowledge of disease-regulating circuits and accelerated the development of technological breakthroughs in molecular interrogation methods. As additional lncRNAs were discovered to be associated with cardiovascular physiology, critical concerns remained unresolved before we could fully exploit the therapeutic potential of these new biological modulators. On the other hand, the mechanism by which lncRNAs are targeted to specific genomic loci remains unknown. Indeed, the temporal-spatial specificity of lncRNA effects is particularly perplexing in light of the fact that a substantial number appear to interact with highly promiscuous variables, such as PRC2 (polycomb repressive complex 2). The biochemical and structural reason for retaining specific lncRNAs that mimic mRNAs in the nucleus is also unknown. Finally, a critical bottom-line issue is: Can we utilize lncRNA’s diagnostic or therapeutic potential to enhance human health? Although this has been a difficult aim to achieve, promising translational initiatives are underway.
